# Assessing accuracy of BiliPredics algorithm in predicting individual bilirubin progression in neonates—results from a prospective multi-center study

**DOI:** 10.3389/fdgth.2025.1497165

**Published:** 2025-02-18

**Authors:** Britta Steffens, Gilbert Koch, Corinna Engel, Axel R. Franz, Marc Pfister, Sven Wellmann

**Affiliations:** ^1^ Pediatric Pharmacology and Pharmacometrics, University of Basel Children’s Hospital (UKBB), Basel, Switzerland; ^2^ Research and Development, NeoPredics AG, Basel, Switzerland; ^3^ Center for Pediatric Clinical Studies (CPCS) Tübingen, University Children’s Hospital Tübingen, Tübingen, Germany; ^4^ Department of Clinical Research, University of Basel, Basel, Switzerland; ^5^ Department of Neonatology, Hospital St. Hedwig of the Order of St. John of God, University Children’s Hospital Regensburg (KUNO), University of Regensburg, Regensburg, Germany

**Keywords:** bilirubin, hyperbilirubinemia, neonatal jaundice, mathematical model, pharmacometrics

## Abstract

**Background:**

Neonatal jaundice affects more than half of neonates. As bilirubin values usually peak few days after hospital discharge, jaundice remains a leading cause of rehospitalization. The recently developed BiliPredics algorithm, integrated in the first CE-approved bilirubin prediction tool, predicts individual bilirubin progression for up to 60 h into the future. Goal of the prospective study was to assess accuracy of this algorithm in predicting individual bilirubin prior to hospital discharge in neonates.

**Methods:**

A prospective multi-center study was conducted in 2021 at the University Children's Hospitals in Tübingen and Regensburg, Germany. Various scenarios differing in type and number of bilirubin measurements and in prediction horizon were tested. Primary objective was prediction accuracy of the BiliPredics algorithm based on total serum bilirubin (TSB) measurements or based on transcutaneous bilirubin (TcB) measurements alone. Secondary objective was prediction accuracy based on combinations of TSB and TcB measurements. For assessment of accuracy, two validation metrics, absolute prediction error (aPE) and relative prediction error (rPE), and two clinical acceptance conditions, margin of error of the 95%-confidence interval (95%-CI) and percentage of clinically relevant mis-predictions defined as aPE>85μmol/L, were investigated.

**Results:**

Out of 455 enrolled neonates, 276 neonates met bilirubin inclusion criteria and were included in the analyses. Irrespective from tested prediction horizons, median rPE was small (8.5% to 9.5%) utilizing TSB measurements for up to 30 and 60 h and slightly higher (13.8%) utilizing TcB measurements for up to 48 h. The same applied for median aPE. Both clinical acceptance conditions were fulfilled across tested scenarios. Results for combined TSB-TcB scenarios up to a prediction horizon of 48 h without adjustment for type of measurement were comparable to TSB and TcB scenarios fulfilling both clinical acceptance conditions.

**Conclusion:**

Results from this prospective study in neonates confirm that the BiliPredics algorithm accurately predicts bilirubin progression up to 60 h with TSB measurements and up to 48 h with TcB or combined TSB-TcB measurements. As such, prediction tools utilizing this algorithm are expected to facilitate and safely optimize jaundice risk assessment at hospital discharge with the potential to reduce jaundice-related rehospitalizations.

## Introduction

Neonatal jaundice or neonatal hyperbilirubinemia is a common condition affecting more than half of all neonates. While elevated bilirubin levels are typically transient and resolve without intervention (1), severe forms of jaundice require prompt therapy, i.e., phototherapy, as they can lead to permanent and irreversible neurological damage including auditory dysfunction and kernicterus (2). To identify neonates at risk for bilirubin-related toxicity, appropriate screening for hyperbilirubinemia is recommended both after birth and before discharge. In numerous countries, neonates are discharged within 48 h after birth which means that peak bilirubin concentrations often only occur after hospital discharge. As a result, hyperbilirubinemia remains a leading cause of rehospitalization during the first year of life (3, 4).

Quantification of bilirubin is crucial for early diagnosis and timely treatment of neonatal jaundice. Despite the advantages of handheld point-of-care devices, laboratory-based total serum bilirubin (TSB) quantification remains the gold standard for bilirubin measurement in blood (5). Since blood bilirubin concentrations correlate well with bilirubin accumulation in the skin, transcutaneous bilirubin (TcB) quantification is widely used as a non-invasive and reliable method for estimating TSB levels with high sensitivity (6).

Currently, the management and prevention of neonatal hyperbilirubinemia in clinical practice relies on static, gestational age-dependent nomograms, which are based on bilirubin percentiles (1). These nomograms are population-based and classify neonates into risk groups without accounting for individual patient characteristics and bilirubin kinetics. Over the past decade, enhanced risk stratification approaches have been developed to predict hyperbilirubinemia both after birth (7) or before discharge (8) by incorporating additional clinical factors. Recently, the American Academy of Pediatrics (AAP) published a revised clinical practice guideline for the management of hyperbilirubinemia in neonates ≥35 weeks of gestation (9). Among the key action statements is the calculation of the bilirubin rate of increase (per hour) based on consecutive bilirubin measurements, to identify neonates at higher risk for severe hyperbilirubinemia. However, despite established guidelines and high healthcare standards, noncompliance by healthcare professional still contributes to preventable cases of kernicterus (10–12). Therefore, there is a need for accurate prediction of the individual bilirubin progression beyond hospitalization, i.e., beyond hospital discharge, based on just a few bilirubin measurements taken after birth, to help to prevent severe hyperbilirubinemia and reduce the need for rehospitalizations.

The recently developed pharmacometrics-based (PMX-based) BiliPredics algorithm, formerly known as NeoPrediX B.1, enables the prediction of individual bilirubin progression for up to 60 h into the future for the first time (13). The algorithm utilizes a PMX model structure that incorporates physiological principles, such as the perturbation of bilirubin production and elimination due to maturation processes (13–17). Integrated into the first CE-approved bilirubin prediction tool, this PMX-based algorithm utilizes one or more bilirubin values measured after birth along with a few clinically relevant patient characteristics, such as gestational age (GA), birth weight, and delivery mode. Here, for predicting the individual bilirubin progression, a non-linear mixed effects approach (estimating both fixed and random effects) combined with Empirical Bayesian Estimation is applied (18). All details of the PMX model have been previously disclosed in a publicly accessible patent (19).

Unlike traditional nomogram-based risk classifications, the BiliPredics algorithm not only assesses the current status but provides a prediction of the individual time course of bilirubin levels over a specified time horizon, e.g., up to 60 h into the future. This unique feature allows for easy integration of the BiliPredics algorithm (or the CE-approved BiliPredics tool, respectively) into the clinical workflow, supporting clinical decisions related to the timing of phototherapy, hospital discharge, and optimizing neonatal care at home.

A prospective study was conducted at the University Children's Hospitals in Tübingen and in Regensburg, Germany. The primary aim was to investigate accuracy of the BiliPredics algorithm and to validate its performance on an independent external dataset. Various levels of validation were explored, including different bilirubin measurement types (TSB and TcB alone or in combination) and different prediction horizons ranging from 30–60 h. The primary outcome was prediction accuracy based on “pure” TSB or “pure” TcB scenarios up to 30 and 60 h, or 48 h, respectively. As secondary outcome, accuracy was assessed for scenarios involving combinations of any two or any three bilirubin measurements (TSB and/or TcB) up to 48 h. In addition, findings were compared with those from a previously retrospective study conducted in Regensburg, Germany (13).

## Methods

### Study design and data collection

A prospective multi-center observational study led by the Center for Pediatric Clinical Studies (CPCS) Tübingen, Germany, was conducted at two University Children's Hospitals in Germany, namely the University Children's Hospital Tübingen and the University Children's Hospital (KUNO) Regensburg. Eligible neonatal patients were recruited between August 9th, 2021, and November 29th, 2021. This study followed the Consolidated Standards of Reporting Trials (CONSORT) guidelines (20).

Neonatal patient data were eligible if GA at birth was at least 34 completed weeks, if the first bilirubin measurement was performed not later than 72 h after birth, and if parental consent was given. Study subjects were excluded in case of birth weight <1,500 g, phototherapy or exchange transfusion prior to inclusion, genetically defined syndrome or severe congenital malformation adversely affecting life expectancy, admission for *a priori* planned palliative care, or if parents were not fluent in German (below B1 level). The exclusion of such neonates with very low birth weight (<1,500 g) was due to the fact that the BiliPredics algorithm had not been trained on such data. At both study sites, TSB levels were measured using validated standard photometric assays, such as the Bilimeter 3D (Pfaff Medical GmbH, Germany). TcB levels were measured using a transcutaneous optical device (JM-105 device, Dräger Medical GmbH, Germany).

The study was approved by the research ethics boards of the University of Tübingen and of Regensburg and is registered at Clinicaltrials.gov (NCT05121311). Written informed consent was obtained from parents or guardians of all participants before inclusion. Prospectively collected data were captured in state-of-the-art electronic data base, secuTrial®.

### Statistical analysis methods for validation

#### Definition of validation metrics and performance criteria

Two validation metrics [see (13)], and two types of validation criteria were defined to validate performance accuracy. The idea was to compare the predicted bilirubin measurement $B_{\mathrm{pred}}$ to the observed bilirubin measurement $B_{\mathrm{obs}}$ at a certain time point in terms of the absolute prediction error aPE and the percental relative prediction error rPE for each scenario. Based on the **prediction error** PE, i.e.,

$$PE=\;B_{\mathrm{pred}}-\;B_{\mathrm{obs}},$$

we defined the **absolute prediction error** aPE as the absolute value of the prediction error PE, i.e.,

$$aPE\;=|\;B_{\mathrm{pred}}-B_{\mathrm{obs}}|,$$

and the **relative prediction error (in %)** rPE as relating the absolute prediction error aPE to the observed bilirubin measurement $B_{\mathrm{obs}}$, i.e.,

$$rPE\;=\frac{\left|\;B_{\mathrm{pred}}-B_{\mathrm{obs}}\right|}{B_{\mathrm{obs}}}\;.$$

**Figure 1:**
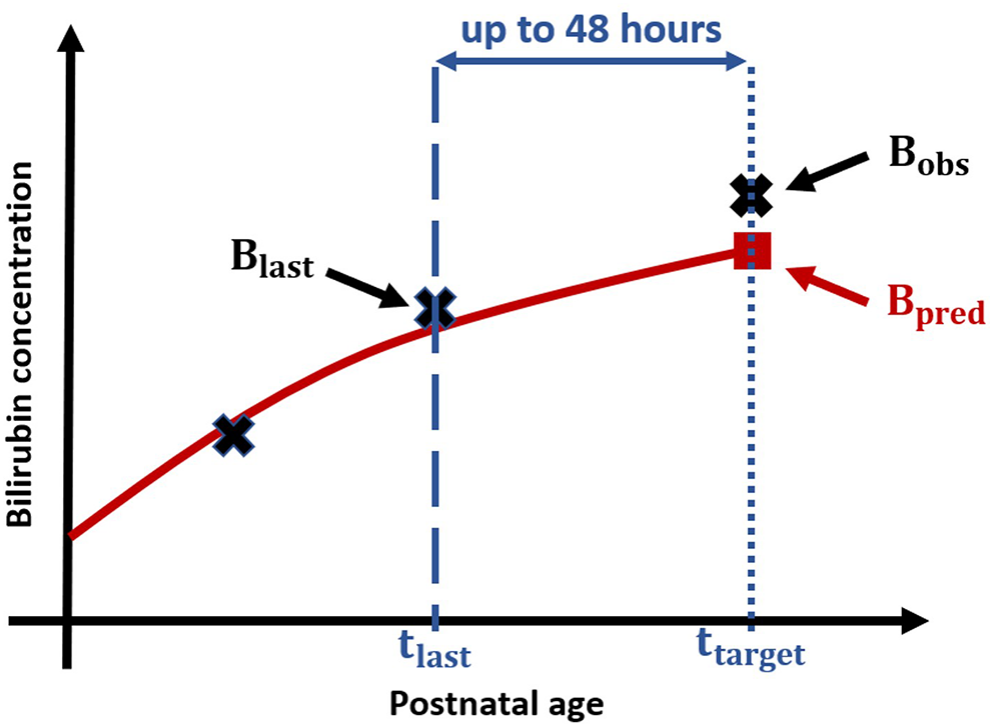
Experimental setup for performance validation: Black crosses display measured Bilirubin values, blue dashed line corresponds to PNA $t_{\mathrm{last}}$ at which the second bilirubin measurement $B_{\mathrm{last}}$ is taken and the blue dotted line to the PNA $t_{\mathrm{target}}$ of target bilirubin measurement $B_{\mathrm{obs}}$ resp. $B_{\mathrm{pred}}$, and the blue arrow represents the time difference between last measurement and target measurement, i.e., the prediction horizon. The red solid line visualizes the (based on the first two crosses) predicted bilirubin progression and the red square displays the predicted bilirubin value.

Figure 1 displays the experimental setup for applied performance validation, as example for a prediction based on 2 bilirubin measurements and a prediction horizon of 48 h.

For validating the performance of the algorithm, two types of validation criteria were defined, namely a clinical acceptance criterion and a more stringent exactness criterion. Both criteria are based on the maximal range of clinically relevant mis-prediction determined to be 85 *µ*mol/L by healthcare professionals. The value of 85 *µ*mol/L represents the difference between GA-specific phototherapy thresholds and exchange transfusion thresholds as defined in the revised 2022 AAP clinical practice guidelines (9).

**Clinical acceptance criterion:** In these clinically relevant criterion, accuracy of the prediction is considered clinically acceptable if

(i) the margin of error of the 95%-confidence interval of prediction errors is between ±85 *µ*mol/L
(ii) 95% of all absolute prediction errors are not exceeding 85 *µ*mol/L

For additional “pressure” testing of the BiliPredics algorithm two conditions for a theoretical, more stringent exactness criterion were defined.

**Exactness criterion:** With this more stringent and conservative criterion, accuracy of the prediction is considered exact and high enough if

(i) the margin of error of the 95%-confidence interval of prediction errors is between ±70 *µ*mol/L
(ii) no absolute prediction error exceeds 85 *µ*mol/L

In both cases, the statistical 95% CI-condition is based on applying the Bland-Altman method involving mean and standard deviation of the prediction errors (21).

Note that from a clinical perspective, under-prediction of actual bilirubin level is clinically relevant since it possibly results in missing out of neonates at risk for hyperbilirubinemia.

#### Definition of validation scenarios

The BiliPredics algorithm is capable of handling a variety of input data regarding type and number of bilirubin measurements and provides different prediction time horizons as output. For performance validation, we defined various clinically relevant validation scenarios, differing in the type of bilirubin measurements (TSB and/or TcB), the number of bilirubin measurements (1, 2 or 3 serial measurements), and the appropriate prediction time horizons (30, 48 or 60 h).

In line with the input criteria implemented in the CE-approved BiliPredics tool, the following input rules were applied for defining validation scenarios: (i) the time point of the 1st TSB measurement must be between 8 and 72 h of postnatal age (PNA), (ii) additional time points of TSB/TcB measurements must be between 24 and 96 h of PNA, and (iii) the time difference between two subsequently performed bilirubin measurements must be at least 8 h of PNA.

Main aim of this research article is to assess the performance accuracy of the BiliPredics algorithm based on “pure” TSB or TcB scenarios, i.e., scenarios with one type of measurement only (either TSB or TcB measurements). The three “pure” TSB or TcB scenarios are collectively referred to as Population A:.

**Scenario 1 (one TSB measurement, 30 h)**: Prediction of bilirubin progression up to 30 h based on one TSB measurement
**Scenario 2 (two TSB measurements, 60 h)**: Prediction of bilirubin progression up to 60 h based on two TSB measurements
**Scenario 3 (three TcB measurements, 48 h)**: Prediction of bilirubin progression up to 48 h based on three TcB measurements
As secondary aim, and as an extension of aforementioned three “pure” TSB or TcB scenarios, validation was performed on scenarios with combinations of any two or any three bilirubin measurements (i.e., scenarios with TSB, TcB, or combined TSB-TcB measurements), with a prediction horizon of up to 48 h. In the following, these combined TSB-TcB scenarios are collectively referred to as Population B:
**Scenario 4 (any two TSB-TcB measurements, 48 h):** Prediction of bilirubin progression up to 48 h based on any combination of two bilirubin measurement (i.e., scenario with two TSB, TcB, or combined TSB-TcB measurements).
**Scenario 5 (any three TSB-TcB measurements, 48 h)**: Prediction of bilirubin progression up to 48 h based on any combination of three bilirubin measurements (i.e., scenario with three TSB, TcB, or combined TSB-TcB measurements).

For validating the two combined TSB-TcB scenarios (Scenarios 4 and 5) the necessary datasets were generated according to the following additional input rules. Please note that for these datasets the input regarding time point of bilirubin measurements was extended to 120 h instead of 96 h.

• The first bilirubin measurement is chosen as the first measurement per patient taken between 8 and 72 h
• The second bilirubin measurement is chosen as the first subsequent measurement taken at least 8 h after the first measurement and between 24 and 120 h
• For Scenario 5, with any three arbitrary bilirubin measurements, the third bilirubin measurement is chosen as the first subsequent measurement taken at least 8 h after the second measurement and between 24 and 120 h
• The measurement that is to be predicted is chosen as the one taken at least 8 h after the second/third measurement and between 24 and 120 h, and being the last measurement taken within the predefined prediction horizon
• For additional “pressure” testing, in case of two bilirubin measurements collected at the same time, TcB is chosen, due to its higher variability

Known difference between TcB and TSB measurements was quantified by Taylor et al. (2015) (22) as a mean difference of 14.3 *µ*mol/L. Accounting for this known difference, we harmonized the measurements in these two combined scenarios by adjusting all bilirubin measurements depending on the type of the target measurement, i.e., the measurement to be predicted, compare Figure 1.

#### Descriptive analysis of study Population A

A descriptive analysis was performed for Population A. For the analysis, neonatal patient characteristics such as PNA (hours), body weight (gram, g), GA (weeks), sex, and delivery mode as well as bilirubin-related clinical and laboratory data such as type of bilirubin measurements, the first and the target bilirubin measurement together with PNA at first and at target bilirubin measurement, were considered. Categorical items are expressed as number and percent, continuous items are presented as median and interquartile range (IQR).

### Comparison with previous retrospective study

A retrospective study was conducted at the University Children's Hospital (KUNO) Regensburg, Germany, to externally validate PMX-based algorithm. In this study, validation performance of the algorithm was investigated for solely TSB scenarios with prediction horizons up to 30 h (one TSB measurement) and up to 60 h (two or two and more TSB measurements) (13) based on the median aPE and the median rPE. All TSB measurements were performed as total bilirubin utilizing a Bilimeter 3D (Pfaff Medical GmbH, Germany). For these retrospective data we additionally applied the clinical acceptance and the more stringent exactness criterion, and compared results to those for Population A, Scenarios 1 and 2.

### Three additional validation analyses regarding adjusting for type of measurement, hemolytic disease, and rate of bilirubin increase

In addition, three clinically relevant validation analyses were performed to (i) compare results with and without adjusting for type of bilirubin measurement (TSB or TcB measurements), (ii) investigate a subgroup of patients with hemolytic disease, and (iii) predict the rate of bilirubin increase as recommended by the revised AAP guidelines (9).

#### Validation of algorithm performance without accounting for type of bilirubin measurement

As stated in the revised AAP guidelines (9) and discussed in various studies, direction and magnitude of TSB-TcB differences not only decrease with increasing TSB level (22, 23) but may also depend on skin melanin concentration (24, 25), and the devices utilized for measuring TcB levels (6, 26–28). Aiming to reduce complexity of the clinically useful BiliPredics algorithm, we re-validated (applying the same metrics and criteria) the performance of all combined TSB-TcB scenarios (Scenarios 4 and 5) without adjusting any bilirubin measurements used for prediction. These new results were compared to those obtained for Population B.

#### Validation of algorithm performance in subgroup of patients with hemolytic disease

A clinically relevant subgroup of Population A, patients with hemolytic disease (including blood group incompatibility), was analyzed separately. The results of this sensitivity analysis were compared to those from primary analysis with Population A.

#### Validation of algorithm performance in the context of predicting rate of bilirubin increase

As stated in the revised 2022 AAP guidelines (9), key action statement 7, the rate of bilirubin increase is a key parameter to assess the risk for hyperbilirubinemia. Since the Bilipredics algorithm predicts the time course of bilirubin progression, i.e., the bilirubin value at target $B_{\mathrm{pred}}$, the algorithm can also be applied to predict the rate of bilirubin increase per hour (mg/dl per hour) for the predicted target $B_{\mathrm{pred}}$, and the last measurement before prediction $B_{\mathrm{last}}$. This rate of increase is calculated as follows:

$$\mathrm{incrRate}=\frac{B_{\mathrm{target}}-B_{\mathrm{last}}}{t_{\mathrm{target}}-t_{\mathrm{last}}}$$

where $t_{\mathrm{target}}$ is the time point (in hours) at which $B_{\mathrm{target}}$, i.e., $B_{\mathrm{obs}}$ or $B_{\mathrm{pred}}$, was taken, and $t_{\mathrm{last}}$ is the time point (in hours) at which $B_{\mathrm{last}}$ was taken (compare Figure 1).

### Statistical data analysis, data presentation, and applied software packages

Completeness and correctness of all data was checked prior to all analyses. In case of missing values, no data imputation was performed, and patients were excluded. For Population A, analyses regarding validation and all descriptive analyses have independently been done by Dr. Corinna Engel from the Center for Pediatric Clinical Studies (CPCS) Tübingen, Germany, using SAS 9.4, and results were documented in a NeoPredics-internal Statistical Analysis Report. NeoPredics AG had no access to study data until all data were captured and data analysis was completed. After a safe data transfer, Dr. Britta Steffens performed *post-hoc* analyses regarding Population B and all additional validation analyses, and repeated analyses for Population A for generating tables and figures. These analyses were done in R 4.1.0 (R core team, Vienna, Austria).

## Results

### Descriptive analysis of study population

**Figure 2:**
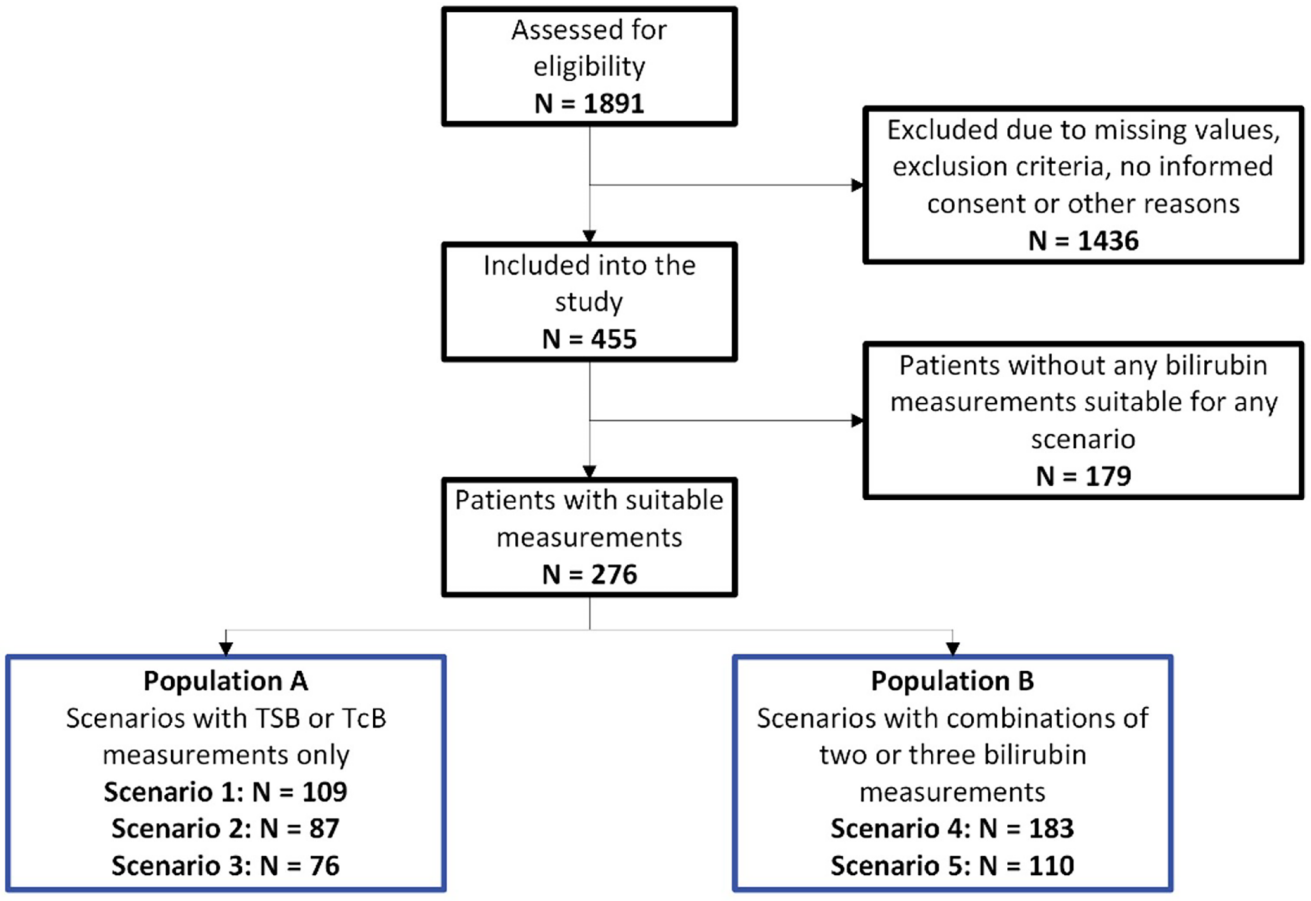
CONSORT graph: CONSORT graph and composition of the overall population (*N* = 276) and the two study populations, Population A and Population B.

This prospective multi-center observational study enrolled 455 study subjects after applying inclusion and exclusion criteria. A total of 276 study subjects met bilirubin input criteria and thus, were included in the study. The scheme in Figure 2 displays the CONSORT graph for the composition of the different scenario populations and provides information on the number of excluded patients. It should be noted that several neonatal patients were suitable for more than one scenario and, accordingly, the total number of patients in the different scenarios exceeded the *N* = 276 study subjects.

Descriptive analysis was performed for all subjects included in the study with a total of *N* = 276 individual neonates that met bilirubin input criteria. Table 1 summarizes results for GA (weeks), sex (% female), birth weight (g), delivery mode (% cesarian section), first TSB or TcB measurement provided for prediction (*μ*mol/L) and TSB or TcB measurement at target (μmol/L), and for PNA (days) at first bilirubin measurement provided for prediction and at target.

**Table 1:** Patient characteristics of overall number of patients included in the study (*N* = 276): Continuous variables are given as median (IQR), categorical variables are given as number (%).

Type of measurements	TSB measurements		TcB measurements
	One TSB measurement (*N* = 142)	Two TSB measurements (*N* = 87)	Three TcB measurements (*N* = 125)
Gestational Age (weeks)	38.7 (37.0, 39.9)	38.1 (35.9, 39.9)	39.3 (38.3, 40.4)
Sex (% female)	55 (38.7%)	34 (39.1%)	56 (44.8%)
Birth Weight (g)	3,155 (2,670, 3,510)	3,100 (2,630, 3,535)	3,300 (3,050, 3,600)
Delivery mode (% c-section)	50 (35.2%)	28 (32.2%)	46 (36.8%)
PNA at first bilirubin measurement (h)	45 (33, 53)	39 (29, 49)	21 (14, 30)
First bilirubin measurement (*µ*mol/L)	165.9 (128.3, 203.5)	167.6 (95.8, 194.9)	102.6 (68.4, 128.3)
PNA at target (h)	69 (52, 79)	102 (84, 120)	98 (78, 115)
Bilirubin measurement at target(µmol/L)	208.6 (153.9, 246.2)	237.7 (186.4, 278.7)	191.5 (160.7, 220.6)

### Performance validation of BiliPredics algorithm

In this paragraph, the results regarding performance validation of the BiliPredics algorithm for Population A with “pure” TSB or TcB scenarios and Population B with combined TSB-TcB scenarios, distinguished by scenario each, are presented.

#### Performance validation for Population A

Table 2 presents distributions of the prediction errors per scenario, expressed in terms of the margin of error of 95%-CI as well as the median [IQR] of relative PE (rPE) and the number (%) of mis-predictions. Across all scenarios, the rPE ranged from 8%–14%. As expected, both the aPE and rPE were slightly higher in Scenario 3, which utilized TcB measurements, compared to those utilizing TSB measurements (Scenarios 1 and 2), despite the longer prediction horizon in Scenario 2.

**Table 2:** Summary of results for Population A, Scenarios 1–3: Results for median (together with IQR) of aPE and rPE, margin of error of 95% CI, and number (%) of clinically relevant over- and under-predictions.

	Type/Number of measurements and prediction horizon	Margin of error of 95% CI	Median (IQR) of rPE	Number (%) of clinically relevant over- and under-predictions	
Scenario 1	One TSB measurement up to 30 h	60.2 *µ*mol/L	8.5% (3.8, 15.2) %	PE<−85μmol/L	1 (0.9%)
(*N* = 109)				PE>+85μmol/L	1 (0.9%)
Scenario 2	Two TSB measurements up to 60 h	73.2 *µ*mol/L	9.5% (4.6, 17.2) %	PE<−85μmol/L	1 (1.2%)
(*N* = 87)				PE>+85μmol/L	3 (3.5%)
Scenario 3	Three TcB measurements up to 48 h	75.9 *µ*mol/L	13.8% (7.5, 26.6) %	PE<−85μmol/L	0 (0%)
(*N* = 76)				PE>+85μmol/L	4 (5.3%)

Regarding number of mis-predictions, all scenarios showed close to 95% (94.7% in Scenario 3) or more than 95% of aPE within the clinically relevant area of ±85μmol/L (Scenarios 1 and 2). Scenarios utilizing TSB measurements (Scenarios 1 and 2) had only about 1.0% of cases with a potentially clinically relevant under-prediction of <−85μmol/L, whereas no clinically relevant under-prediction was observed in Scenario 3 utilizing TcB measurements. A detailed summary of the results, including mean, standard deviation, and median [IQR] of absolute prediction error (aPE), is provided in Table S1 in the Supplementary Material.

The 95% CI clinical acceptance condition was met across all “pure” scenarios. Moreover, for Scenario 1, utilizing only one TSB measurement and a prediction horizon of 30 h, the more stringent 95% CI exactness condition was met as well. For Scenarios 2 and 3, where the prediction horizon was up to 60 and 48 h, respectively, the margin of error was only slightly larger than the required 70 *µ*mol/L (73.2 and 75.9 *µ*mol/L, respectively). Results regarding the clinical acceptance conditions are presented in Table S2 in the Supplementary Material.

#### Performance validation for Population B

Table 3 summarizes the results on the distribution of the prediction errors in terms of the median of rPE, margin of error of 95% CI of PE, and the number (%) of mis-predictions with respect to a aPE of 85 µmol/L. The median of rPE in combined TSB-TcB scenarios, utilizing either two or three arbitrary bilirubin measurements, was of the same order as in the “pure” scenarios (Population A, Scenarios 1–3). As expected, prediction up to 48 h based on three arbitrary bilirubin measurements resulted in lower rPEs, smaller margins of error and fewer (if any) clinically relevant under-predictions. A detailed summary of the results, including mean, standard deviation, and median [IQR] of aPE, is provided in Table S3 in the Supplementary Material.

**Table 3:** Summary of results for Population B, Scenarios 4 and 5, with adjusting for type of measurement: Results for median (together with IQR) of aPE and rPE, margin of error of 95% CI, and number (%) of clinically relevant over- and under-predictions.

	Type/Number of measurements	Margin of error of 95% CI	Median (IQR) of rPE	Number (%) of clinically relevant under- and over-predictions	
Scenario 4	Any two bilirubin measurements	73.4 *µ*mol/L	11.2% (5.3, 21.3) %	PE<−85μmol/L	2 (1.1%)
*N* = 183				PE>+85μmol/L	2 (1.1%)
Scenario 5	Any three bilirubin measurements	64.1 *µ*mol/L	10.6% (5.2, 20.6) %	PE<−85μmol/L	0 (0%)
*N* = 110				PE>+85μmol/L	1 (0.9%)

In terms of clinical acceptance, all scenarios met both conditions. This is consistent with the prediction accuracy observed in the “pure” scenarios in Population A. Moreover, regarding the more stringent exactness criterion, Scenario 5 met the 95% CI condition, whereas in Scenario 4, utilizing two arbitrary bilirubin measurements, the margin of error slightly exceeded the tolerable threshold (73.4 *µ*mol/L compared to 70 *µ*mol/L). Results regarding the clinical acceptance conditions are presented in Table S4 in the Supplementary Material.

### Comparison with previous retrospective study

Results of median of aPE and rPE as well as number (%) of clinically relevant mis-predictions from prospective study are consistent with those the from retrospective study (13). For Scenario 2 with two TSB measurements and a prediction up to 60 h, accuracy was even better with lower median of aPE and rPE and lower percentage of clinically relevant under-predictions. A detailed summary of the results is given in the Supplementary Material, Tables S5 and S6.

### Three additional validation analyses regarding adjusting for type of measurement, hemolytic disease, and rate of bilirubin increase

#### Validation of algorithm performance without accounting for type of bilirubin measurement

For validation of the BiliPredics algorithm without adjusting for type of measurement, we observed a margin of error of 95% CI, rPE, and aPE of the same order as shown in Table 4 when adjusting for the type of measurement (Population B). In addition, the clinical acceptance and the more stringent exactness criteria were met along the same lines as stated in the previous paragraph.

**Table 4:** Summary of results for Population B, Scenarios 4 and 5, without adjusting for type of measurement: Results for median (together with IQR) of rPE, margin of error of 95%-CI, and number (%) of clinically relevant over- and under-predictions.

	Type/Number of measurements	Margin of error of 95%-CI	Median (IQR) of rPE	Number (%) of clinically relevant under- and over-predictions	
Scenario 4	Any two bilirubin measurements	74.6 *µ*mol/L	11.2% (4.9, 20.8) %	PE<−85μmol/L	3 (1.6%)
*N* = 183				PE>+85μmol/L	2 (1.1%)
Scenario 5	Any three bilirubin measurements	64.5 *µ*mol/L	11.3% (5.4, 20.4) %	PE<−85μmol/L	0 (0%)
*N* = 110				PE>+85μmol/L	1 (0.9%)

#### Validation of algorithm performance in subgroup of patients with hemolytic disease

For the clinically relevant subgroups of Population A, patients with hemolytic disease, the more stringent exactness as well as the clinical acceptance criteria were met across all scenarios except for the mis-prediction exactness condition in Scenario 1 with a single patient showing a clinically relevant under-prediction of −94 *µ*mol/L.

#### Validation of algorithm performance in the context of predicting rate of bilirubin increase

For Scenarios 4 and 5, both with and without adjusting for type of measurement, the observed and predicted rates of bilirubin increase are summarized in Table 5 [presented as median (IQR)]. Across all scenarios, i.e., regardless of prediction horizon, as well as number and type of measurements, the observed rates of bilirubin increase closely aligned with those obtained from predicted bilirubin values.

**Table 5:** Summary of observed and predicted bilirubin increase rates for Population B: Increase rates are shown for Scenarios 4 and 5, with and without adjusting for type of measurement, given as median (IQR).

	Type/Number of measurements	Adjustment for type of measurement	Observed bilirubin increase rate	Predicted bilirubin increase rate
Scenario 4	Any two bilirubin measurements	With adjustment	0.07 (0.03, 0.12) mg/dl per hour	0.07 (0.04, 0.1) mg/dl per hour
*N* = 183		Without adjustment	0.07 (0.02, 0.12) mg/dl per hour	0.07 (0.04, 0.1) mg/dl per hour
Scenario 5	Any three bilirubin measurements	With adjustment	0.05 (0.002, 0.09) mg/dl per hour	0.06 (0.03, 0.09) mg/dl per hour
*N* = 110		Without adjustment	0.04 (0.007, 0.08) mg/dl per hour	0.06 (0.03, 0.09) mg/dl per hour

## Discussion

In this section, we discuss key findings derived from analyses using data from a prospective multi-center study in neonates. We had two objectives: First, to validate prediction accuracy in scenarios based on either “pure” TSB or TcB measurements or on any combination of at least two bilirubin measurements (TSB, TcB, or combined TSB-TcB) with different prediction horizons, and second, to compare these findings with results from a retrospective study (13).

For Population A (main aim) scenarios utilizing TSB measurements (Scenarios 1 and 2) were associated with slightly better aPE and rPE as compared to those with TcB measurements (Scenario 3). All three scenarios fulfilled the 95% CI condition and the mis-prediction condition of clinical acceptance with only a small number of clinically relevant under-predictions with respect to a PE of 85 *µ*mol/L (1.2% and 1.5% for Scenarios 1 and 2, and 0% for Scenario 3). In addition, the 95% CI more stringent exactness condition was fulfilled for all scenarios except for Scenario 3 with prediction up to 48 h utilizing three TcB measurements where the margin of error of the 95% CI was only slightly larger than the pre-defined threshold (75.9 *µ*mol/L compared to 70 *µ*mol/L). These findings align with the anticipated magnitude of underlying variability associated with TSB measurements in clinical practice (5%–15%) (13, 29) and the greater variability inherent to TcB measurements (30).

Regarding Population B (secondary aim), scenarios with combinations of any two or three TSB and/or TcB measurements were evaluated. These scenarios are clinically relevant as numerous hospitals monitor bilirubin levels in neonates using a combination of TSB and TcB measurements. In these scenarios, the observation period was extended from 96 h–120 h, while the previous version of the BiliPredics tool limited predictions to a PNA of 144 h. As a result, the prediction horizon remained below 48 h, not affecting prediction accuracy. Several publications (22–28) address the discrepancies between the two types of measurements and the accuracy of TcB measurements compared to TSB measurements. Taylor et al. (2015) (22) quantified these discrepancies in a retrospective study. For combined TSB-TcB scenarios (Scenarios 4 and 5), all bilirubin measurements were adjusted based on Taylor's findings, considering the type of bilirubin measurement being predicted. Due to the larger variability and intrinsic measurement error associated with TcB measurements, prediction errors in TSB-TcB scenarios (Scenario 1 and 2) were slightly higher compared to “pure” TSB scenarios but smaller than in the “pure” TcB scenarios (Scenario 3). Results on clinical acceptance and exactness criteria were comparable to Population A. However, in clinical practice, the distinction between TSB and TcB measurements is often overlooked when interpreting bilirubin levels. Additionally, harmonization of these measurements is complex, influenced by factors such as PNA, skin anatomy, bilirubin levels, and variability in available measurement devices (22–28). To address this, a sensitivity analysis was conducted to compare results from combined TSB-TcB scenarios (Population B) without adjusting for the type of measurement. Interestingly, results were consistent across all scenarios indicating that the BiliPredics algorithm no longer requires differentiation between measurement types. This expanded application spectrum, encompassing “pure” TSB and TcB scenarios as well as combined TSB-TcB scenarios, simplifies input rules and enhances the tool's flexibility for diverse clinical settings.

An additional objective of this investigation was to compare results of Scenarios 1 and 2 with those from a previous retrospective validation study using TSB data conducted at the University Children's Hospital (KUNO) in Regensburg, Germany (13). Results for median of aPE, median of rPE and number of mis-predictions were comparable. Notably, Scenario 2, which utilized TSB measurements with prediction horizon up to 60 h, demonstrated fewer clinically relevant under-predictions compared to those reported in retrospective study (Supplementary Material, Tables S5 and S6).

In addition to the sensitivity analysis regarding adjustment for the type of measurement, two clinically relevant validation analyses were performed to further evaluate the usability of the BiliPredics algorithm as a bedside, CE-approved bilirubin prediction tool. First, performance was assessed in a subgroup of patients with hemolytic disease. This subgroup met both the theoretical exactness and the clinical acceptance conditions in all scenarios, except for the mis-prediction exactness condition in Scenario 1 with a single patient showing an under-prediction of −94 *µ*mol/L. Second, in light of the revised 2022 AAP guidelines (9), we compared the predicted rate of bilirubin increase with the actual rate of increase. Across all scenarios and populations, these increase rates were comparable, indicating the algorithm's ability to reliably predict individual bilirubin dynamics—an essential aspect of hyperbilirubinemia risk assessment at hospital discharge. The BiliPredics tool offers several advantages for patient care. First, it integrates seamlessly with patient information systems to automatically plot measured bilirubin values on the relevant AAP graph, enhancing accuracy and reducing manual errors. Second, by predicting the individual bilirubin course up to 30 h based on one TSB or up to 60 h based on two or three subsequent TSB measurements, the tool supports optimal timing of bilirubin monitoring. This functionality is particularly beneficial for less experienced staff, discharge management, reduction of frequency of outpatient visits, thereby minimizing costs and burdens on families. Third, the tool generates user-friendly, easily understandable reports for parents and healthcare providers, further enhancing its practical utility.

This study has some limitations. First, hemolytic diseases in neonates may influence serum bilirubin dynamics and thus, are identified as risk factors for hyperbilirubinemia neurotoxicity in the AAP guideline (9). Second, as Glucose-6-phosphate dehydrogenase (G6PD) deficiency is not part of the nation-wide neonatal screening in Germany, it is unclear whether this condition was present in our study cohort. Additionally, prediction tools are inherently limited by the patient data available at the time of analysis. Emerging conditions, such as dehydration or infection, can significantly alter the bilirubin course. Therefore, clinical evaluation by experienced healthcare providers remains essential even after hospital discharge (9). In essence, the accuracy of prediction tools relies not only on available patient data but also on the clinical judgment and compliance of healthcare professionals.

In conclusion, results from this prospective study in neonates confirm the BiliPredics algorithm's ability to accurately predict bilirubin progression up to 60 h with at least two TSB measurements. Furthermore, high accuracy was found for predicting bilirubin progression up to 48 h based on any combination of at least two TcB or combined TSB-TcB measurements without adjusting for type of bilirubin measurement. As tested scenarios reflect common settings in hospitals, these findings increase usability and applicability of the BiliPredics algorithm in clinical practice. Moreover, the BiliPredics algorithm is expected to facilitate and optimize jaundice risk assessment at hospital discharge, particularly in the context of the continuous trend towards shorter hospitalizations and the revised 2022 AAP jaundice guidelines, ultimately contributing to a reduction in jaundice-related rehospitalizations.

## Data Availability

The datasets presented in this article are not readily available due to patient privacy. Requests for access to the data will be considered by the corresponding author and principal investigators of the included cohorts, and a decision will be made about the appropriateness of the use of the data. If the use is appropriate, a data sharing agreement will be put in place before a fully de-identified version of the dataset used for the analysis with individual participant data is made available. The specific data, documents, and related materials to be shared will be determined during the request process. Requests to access the datasets should be directed to the corresponding author.
