# Supplementary material for: Assessing accuracy of BiliPredics algorithm in predicting individual bilirubin progression in neonates—results from a prospective multi-center study
Source: Front Digit Health. 2025 Feb 18;7:1497165. doi: 10.3389/fdgth.2025.1497165 (PMC11878101; doi:10.3389/fdgth.2025.1497165)
Supplement: Supplementary file 1 [file Table1.docx]

**Supplementary Material**

The Supplementary Material contains detailed results for the primary and the secondary outcome as well as for the additional validation analysis regarding adjustment for type of measurement.

For Population A, Tables S1 and S2 present detailed results regarding validation metrics and clinical acceptance conditions, respectively.

**Table S1**: **Summary of results for Population A, Scenarios 1 to 3:** Results for mean and standard deviation of $PE$, and for median (together with IQR) of absolute prediction error ($aPE$).

| **Population A** | **Prediction horizon** | **Mean** $\boldsymbol{\pm}$ **standard deviation** | **Median [IQR] of** $\boldsymbol{aPE}$ |
| --- | --- | --- | --- |
| Scenario 1  (N = 109) | 30 hours | -3.4 $\pm$ 30.7 µmol/l | 16.6 µmol/  [7.3, 28.8] µmol/l |
| Scenario 2  (N = 87) | 60 hours | 2.6 $\pm$ 37.3 µmol/l | 19.6 µmol/l  [8.5, 41.2] µmol/l |
| Scenario 3  (N = 76) | 48 hours | 17.3 $\pm$ 38.7 µmol/l | 26.4 µmol/l  [13.1, 45.7] µmol/l |

**Table S2:** Summary of results regarding the clinical acceptance criterion for Population A, Scenarios 1 to 3.

|  | **Prediction horizon** | **95%-CI of** $\boldsymbol{PE}$**s**  **between** $\boldsymbol{\pm85}$ **µmol/l** | **95% of** $\boldsymbol{aPE\leq85}$ **µmol/l** |
| --- | --- | --- | --- |
| **Scenario 1** | 30 hours | 🗸 | 🗸 |
| **Scenario 2** | 60 hours | 🗸 | 🗸 |
| **Scenario 3** | 48 hours | 🗸 | - |

For Population B, Tables S3 and S4 present detailed results regarding validation metrics and clinical acceptance conditions, respectively.

**Table S3**: **Summary of results for Population B, Scenarios 4 and 5:** Results for mean and standard deviation of $PE$, and for median (together with IQR) of absolute prediction error ($aPE$).

| **Population B** | **Prediction horizon** | **Mean** $\boldsymbol{\pm}$ **standard deviation** | **Median [IQR] of** $\boldsymbol{aPE}$ |
| --- | --- | --- | --- |
| Scenario 4  (N = 183) | 48 hours | 0.2 $\pm$ 37.4 µmol/l | 23.7 µmol/l  [11.1, 39.4] µmol/l |
| Scenario 5  (N = 110) |  | -6.9 $\pm$ 32.7 µmol/l | 19.6 µmol/l  [8.5, 41.2] µmol/l |

**Table S4:** Summary of results regarding the clinical acceptance criterion for Population B, Scenarios 4 and 5.

|  | **Prediction horizon** | **95%-CI of** $\boldsymbol{PE}$**s**  **between** $\boldsymbol{\pm85}$ **µmol/l** | **95% of** $\boldsymbol{aPE\leq85}$ **µmol/l** |
| --- | --- | --- | --- |
| **Scenario 4** | 48 hours | 🗸 | 🗸 |
| **Scenario 5** |  | 🗸 | 🗸 |
